# Health Outcomes of Patients with Distal Urea Cycle Disorders Detected by Newborn Screening: Data from the Spanish National Registry

**DOI:** 10.3390/ijns12020044

**Published:** 2026-06-18

**Authors:** Raquel Yahyaoui, Pilar Quijada-Fraile, Javier Blasco-Alonso, Inmaculada Vives, David Gil Ortega, Maria-Luz Couce, Paula Sánchez-Pintos, M. Concepción García Jiménez, Silvia Meavilla Olivas, Camila García Volpe, Mariela de los Santos Mercedes, Ángels García-Cazorla, Ana Felipe-Rucián, Lucy Dougherty-de Miguel, Ana Morais López, Ana Bergua Martínez, José David Andrade Guerrero, Sinziana Stanescu, Amaya Belanger, Mercedes Gil-Campos, María José Comino Monroy, Marcello Bellusci, Patricia Pérez-Mohand, Delia Barrio-Carreras, Belén Pérez, Elena Martín-Hernández

**Affiliations:** 1 Laboratorio de Metabolopatías y Cribado Neonatal, Unidad de Diagnóstico y Tratamiento de Enfermedades Metabólicas Hereditarias, Hospital Regional Universitario de Málaga, IBIMA-Plataforma BIONAND, 29011 Malaga, Spain; 2 Departamento de Biomedicina y Odontología, Facultad de Ciencias Biomédicas y Deporte, Universidad Europea de Andalucía, 29010 Malaga, Spain; 3 Unidad de Enfermedades Mitocondriales-Metabólicas Hereditarias, Hospital Universitario 12 de Octubre, Instituto de Investigación Imas12, CIBERER, MetabERN, 28041 Madrid, Spain; pilar.quijadaf@salud.madrid.org (P.Q.-F.); delia.barrio@salud.madrid.org (D.B.-C.);; 4 Departamento de Gastroenterología y Nutrición Infantil, Unidad de Diagnóstico y Tratamiento de Enfermedades Metabólicas Hereditarias, Hospital Regional Universitario de Málaga, IBIMA-Plataforma BIONAND, Universidad de Málaga, 29011 Malaga, Spain; 5 Departamento de Gastroenterología y Nutrición Infantil, Hospital Clínico Universitario Virgen de la Arrixaca, 30120 Murcia, Spain; 6 Unidad de Diagnóstico y Tratamiento de Enfermedades Metabólicas Congénitas, Hospital Clínico Universitario, IDIS, RICORS, CIBERER, MetabERN, 15706 Santiago de Compostela, Spainpaula.sanchez.pintos@sergas.es (P.S.-P.); 7 Unidad de Enfermedades Metabólicas, Hospital Universitario Miguel Servet, 50009 Zaragoza, Spain; 8 Unidad de Enfermedades Metabólicas, Hospital Sant Joan de Déu, MetabERN, 08950 Barcelona, Spain; 9 Hospital Universitario Vall d’Hebrón, Instituto de Investigación VHIR, 08035 Barcelona, Spain; ana.felipe@vallhebron.cat (A.F.-R.);; 10 Departamento de Nutrición Pediátrica, Hospital Universitario La Paz, 28046 Madrid, Spain; 11 Unidad de Enfermedades Metabólicas, Departamento de Pediatría, Hospital Universitario Ramón y Cajal, MetabERN, 28034 Madrid, Spain; 12 Unidad de Metabolismo Infantil, Hospital Universitario Reina Sofía, IMIBIC, UCO, CIBEROBN, 14004 Cordoba, Spain; mercedes_gil_campos@yahoo.es; 13 Unidad de Metabolismo Infantil, Hospital Universitario Reina Sofía, IMIBIC, UCO, 14004 Cordoba, Spain; 14 Centro de Diagnóstico de Enfermedades Moleculares, Universidad Autónoma, IdiPAZ, CIBERER, 28049 Madrid, Spain

**Keywords:** urea cycle disorders, newborn screening, hyperammonemia, argininosuccinate synthetase deficiency, registry

## Abstract

Urea cycle disorders (UCDs) are rare inherited metabolic diseases associated with toxic hyperammonemia, leading to severe neurological damage and early mortality. Early diagnosis of distal UCDs through newborn screening (NBS) enables presymptomatic intervention; however, comparative real-world outcome data remain limited. We conducted a retrospective, multicenter study using data from the Spanish UCD Registry to describe the clinical characteristics and compare health outcomes between patients diagnosed through NBS (*n* = 40) and those diagnosed after clinical presentation (*n* = 53). Patients identified by NBS showed a markedly more favorable clinical prognosis, with a mortality rate of 2.5% compared with 15.1% in the unscreened cohort, as well as significantly lower rates of neurological involvement, fewer hospital admissions due to metabolic decompensation, and a reduced need for liver transplantation. Screening also identified a high prevalence of argininosuccinate synthetase deficiency (ASS1D) cases with attenuated biochemical profiles, highlighting the relevance of sensitive screening cutoffs. These findings provide real-world evidence that presymptomatic diagnosis through NBS is associated with improved survival and long-term neurological outcomes in patients with distal UCDs.

## 1. Introduction

Urea cycle disorders (UCDs) are a group of rare, inherited inborn errors of metabolism caused by deficiencies in the enzymes or transporters required for the detoxification of ammonia into urea [1]. These disorders result in the accumulation of toxic nitrogenous compounds, primarily hyperammonemia, which can lead to irreversible neurological damage, coma, and death, particularly in the neonatal period [2].

The latest International Classification of Inherited Metabolic Disorders (ICIMD) includes 10 genes within the subgroup of hyperammonemias and UCDs [3]. For the purposes of this manuscript, we focus on distal UCDs, defined as those that can be detected with sufficient reliability by newborn screening (NBS): argininosuccinate synthetase deficiency (ASS1D, citrullinemia type I, MIM #215700), argininosuccinate lyase deficiency (ASLD, argininosuccinic aciduria, MIM #207900), arginase deficiency (ARG1D, MIM #207800), and citrin deficiency (mitochondrial aspartate/glutamate carrier deficiency, MIM #605814, #603471).

The collective incidence of UCDs is estimated at 1 in 35,000 live births in the United States [4] and approximately 1 in 36,000 live births in Spain [5], making these disorders a significant concern in pediatric and metabolic medicine, although the estimated incidence varies among deficiencies [5]. Clinical presentation is highly variable, ranging from a severe neonatal-onset hyperammonemic crisis to a more insidious late-onset course, with some patients remaining asymptomatic [2].

The prognosis of patients with UCDs is primarily influenced by the severity of hyperammonemia, particularly peak plasma ammonia concentration, and by age at onset, with neonatal presentation typically reflecting very low residual enzymatic activity [5,6]. The typical manifestations in newborns with hyperammonemia are nonspecific and may include poor feeding, vomiting, loss of thermoregulation, irritability, and somnolence, which may progress to lethargy and coma [1,2,5,6,7,8]. A recent study in Spain showed that overall mortality for these deficiencies is 14.9%, although this figure varies among specific deficiencies [5]. Therefore, patients presenting with a neonatal hyperammonemic crisis often experience high mortality and significant long-term neurocognitive impairment despite aggressive management. This critical window of vulnerability highlights the need for presymptomatic diagnosis [9].

NBS for distal UCDs, typically performed using tandem mass spectrometry (MS/MS) to measure specific amino acids (e.g., elevated citrulline, arginine, or argininosuccinic acid in dried blood spots), offers the opportunity for presymptomatic intervention [10,11]. Early diagnosis and immediate therapeutic intervention are critical to preventing catastrophic neurological outcomes associated with UCDs. NBS allows early detection of hyperammonemic neonates, thereby preventing altered consciousness and neurological symptoms [12,13].

At present, national NBS programs vary widely across the world. The American College of Medical Genetics has proposed a panel comprising 29 core and 25 secondary conditions, in which ASS1D and ASLD are included among the core conditions, whereas ARG1D is classified as a secondary condition [14]. European guidelines recommend NBS for ASS1D and ASLD but state that the benefits of screening for these disorders still need to be fully evaluated. In contrast, for ARG1D, the available evidence is considered insufficient to justify its inclusion in NBS, partly because arginine levels may remain within the normal range during the first days of life [2]. Despite these recommendations, robust real-world data directly comparing the long-term clinical outcomes and disease characteristics of patients diagnosed through NBS with those of patients diagnosed after symptom onset (the “unscreened” or “symptomatic” cohort) remain limited. Moreover, there is a need to understand how NBS affects the prevalence distribution and biochemical presentation of specific UCD subtypes, such as ASS1D and ASLD, and whether the currently used biochemical cutoffs are optimal for detection.

In Spain, UCDs are not currently included in the common national newborn screening portfolio; however, several regional programs have implemented screening for selected distal UCDs at different time points since 2000, and a formal evaluation process for their potential inclusion at the national level is ongoing.

In this context, a retrospective comparative analysis based on data from the Spanish UCD Registry was conducted to describe the clinical characteristics and evaluate the health outcomes of patients identified through newborn screening and to compare them with those of patients diagnosed after clinical presentation.

## 2. Materials and Methods

### 2.1. Study Design

This observational, retrospective, multicenter study included patients with distal UCDs diagnosed in Spain. The study was conducted by the UCD Study Group of the Spanish Association of Inborn Errors of Metabolism (AECOM).

Eligible participants were patients registered in the Spanish Urea Cycle Disorders Registry from its establishment in 2012 through June 2024, with a confirmed diagnosis of ASS1D, ASLD, ARG1D, or citrin deficiency. Patients were classified into two groups: (i) those diagnosed through newborn screening (NBS), regardless of whether symptoms were present at the time of diagnosis, and (ii) those diagnosed after clinical presentation because NBS had not been implemented in their region of birth at that time.

The study protocol was approved by the Local Ethics Committee of the Instituto de Investigación Hospital Universitario 12 de Octubre (imas12) (protocol number 22/629, approved on 31 January 2023) and by the corresponding Spanish regulatory authority. Approval was also obtained from the local research ethics committees of all participating centers. Written informed consent for participation in the study was obtained from all patients or their legal guardians.

### 2.2. Endpoints and Variables

The primary endpoint of this study was to characterize comprehensively the clinical features and health outcomes of patients with distal UCDs identified through newborn screening. The secondary endpoint was to compare this screened cohort with patients from the same registry who were diagnosed after symptom onset in regions where newborn screening was not available at birth.

Clinical variables (gender, type of UCD, symptoms at detection, biochemical parameters, genetic study, admissions due to decompensation and hyperammonemia, neurological involvement, and mortality) were analyzed and compared with data from 53 patients diagnosed on the basis of symptoms of the same diseases. Hospitalizations were analyzed as rates per year of follow-up to account for differences in observation time between cohorts. Neurological involvement was defined as the presence of developmental delay/intellectual disability, epilepsy, movement disorder, or abnormal non-transient neuroimaging attributable to UCD. Neurological outcome was assessed through neurological examination and clinical follow-up at each participating center. Physicians were specifically asked to report the presence of developmental delay, cognitive impairment/intellectual disability, behavioral disorders, movement disorders, epilepsy, or severe encephalopathy. When formal neuropsychological evaluation was available, standardized neurocognitive assessment tools were used, including the Kauffman, Brunet–Lézine, WISC-V, and WPPSI-IV scales. Overall, 23 patients underwent formal neuropsychological testing. In cases where standardized neuropsychological assessments were not available, cognitive impairment was determined on the basis of the clinical judgment of the metabolic specialist or neuropediatrician and/or the need for additional educational support at school. Because this was a retrospective, multicenter, registry-based study, neurodevelopmental assessment methods were not fully standardized across all participating centers. Metabolic decompensation was defined as an acute episode requiring hospital admission and/or intravenous therapy associated with hyperammonemia and/or catabolic stress. A preventive transplant was defined as transplantation performed in the absence of acute life-threatening decompensation, based on anticipated disease severity and multidisciplinary assessment.

### 2.3. Statistical Analysis

A descriptive analysis of both cohorts was performed using means, standard deviations, medians, and interquartile ranges for quantitative variables, and absolute and relative frequencies for qualitative variables. Student’s *t*-test was used to compare continuous variables between the two groups when the assumptions of normality and homoscedasticity were met; otherwise, the nonparametric Mann–Whitney U test was used. Categorical variables were compared using the chi-square test, and Fisher’s exact test was applied when the sample size was small or the expected frequencies were low. When analysis of the linear association between quantitative variables was required, Pearson’s correlation coefficient was used, and statistical significance was evaluated using the corresponding test. In all comparisons, the null hypothesis assumed no differences between the groups, and decisions to reject or not reject it were based on the *p*-value obtained in each test, with 0.05 as the predefined threshold for statistical significance. All analyses were stratified by type of urea cycle disorder, with special attention to differences in age at diagnosis, clinical evolution, and need for liver transplantation. Data processing and analysis were performed using IBM SPSS Statistics software v29.0.2.0 (IBM Corp., Armonk, NY, USA).

## 3. Results

A total of 40 patients diagnosed through NBS from 10 hospitals in Spain were included. A summary of the most relevant demographic, clinical, biochemical, enzymatic, and genetic characteristics of these patients is provided in Table 1, while the complete patient-by-patient dataset is presented in Supplementary Table S1.

### 3.1. Screened Cohort

**Figure 1:**
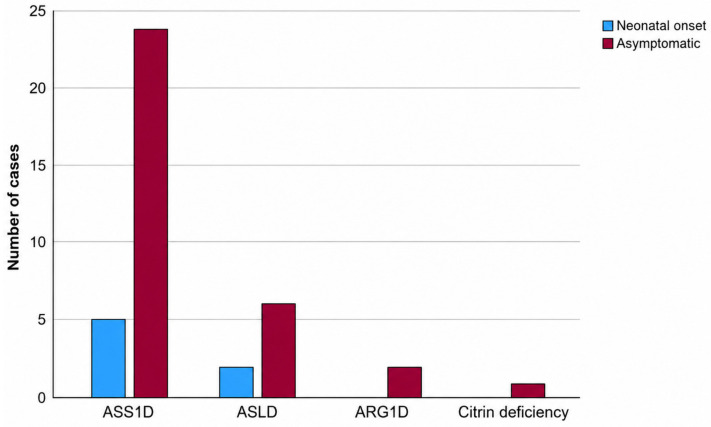
Number of asymptomatic and symptomatic (neonatal onset) cases at detection by UCD type in the newborn screening cohort.

**Figure 2:**
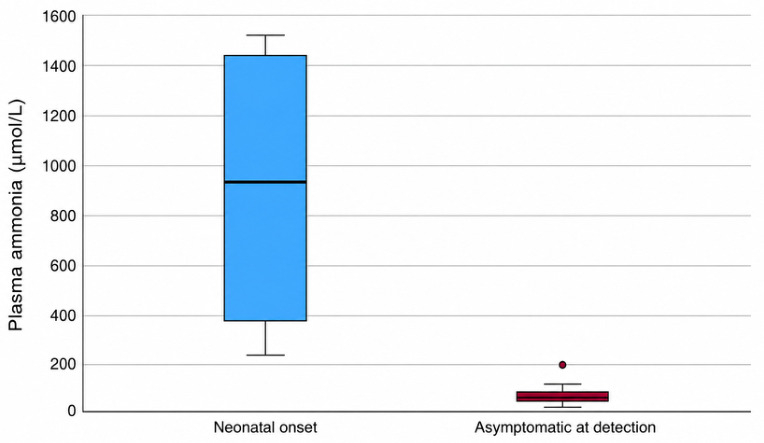
Plasma ammonia concentration at diagnosis in asymptomatic and symptomatic (neonatal onset) cases at detection in the newborn screening cohort during the first week of life.

The screened cohort included 21 males and 19 females: 29 ASS1D (72.5%), 8 ASLD (20%), 2 ARG1D (5%), and 1 citrin deficiency (2.5%). Seven patients had developed symptoms before NBS results became available, whereas the remaining patients were asymptomatic at the time of detection. The mean follow-up was 6.44 ± 4.64 years. The distribution of symptomatic and asymptomatic presentations at diagnosis across the different types of UCDs is shown in Figure 1. Plasma ammonia concentration at diagnosis according to type of presentation are shown in Figure 2. One patient with ASS1D died during the neonatal period in the context of a neonatal-onset metabolic crisis complicated by sepsis (Case 26).

The proportion of ASS1D cases in the screened cohort was higher (72.5%) than in the unscreened cohort (52.8%); however, this difference was not statistically significant (*p* = 0.203). The median citrulline level at screening in this group was 134 µmol/L (IQR 69–446), and the median plasma ammonia concentration at onset was 61.5 µmol/L (IQR 37–136), with 18 of the 29 patients (62%) having dried blood spot (DBS) citrulline levels below 200 µmol/L.

**Figure 3:**
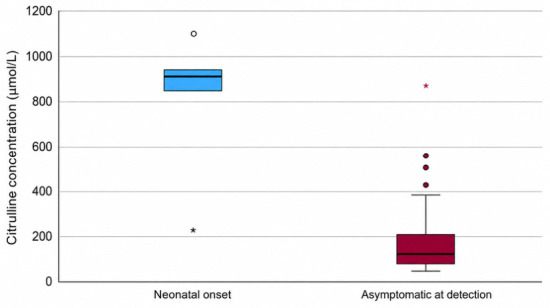
Dried blood spot citrulline concentration in ASS1D cases in the newborn screening cohort according to the presence or absence of symptoms at detection: asymptomatic cases (*n* = 21) versus symptomatic (neonatal onset) cases (*n* = 5).

**Figure 4:**
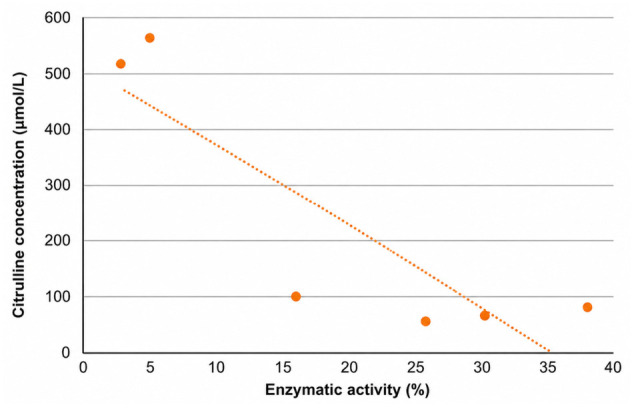
Correlation between enzymatic activity and dried blood spot citrulline concentration in ASS1D-screened patients.

DBS citrulline concentrations in ASS1D cases differed within the newborn screening cohort between asymptomatic and symptomatic (neonatal-onset) patients, as shown in Figure 3, with median values of 116 µmol/L (IQR 71–206) in asymptomatic patients and 903 µmol/L (IQR 850–922) in symptomatic patients. Only the five patients with ASS1D who had symptoms before the screening result showed plasma ammonia concentration above 200 µmol/L at diagnosis. In six patients, ASS1 enzyme activity could be determined in fibroblasts and showed a negative correlation with citrulline concentrations at newborn screening (r = −0.92; *p* = 0.026). This inverse relationship between enzymatic activity and citrulline concentrations in DBS is illustrated in Figure 4.

The proportion of ASLD cases in the screened cohort was slightly lower than in the unscreened cohort (20% vs. 30.18%). The median citrulline level at screening was 103 µmol/L (IQR 48–210), and the median plasma ammonia concentration at onset was 50 µmol/L (IQR 33–125). Only 3 of the 8 cases had argininosuccinic acid measured in DBS at newborn screening, with levels ranging from 103 to 217 µmol/L (reference value < 0.48 µmol/L).

In addition, the two ARG1D cases identified in the newborn screening cohort, both asymptomatic at detection, showed moderately elevated arginine levels in dried blood spots (103 and 196 µmol/L). The case with citrin deficiency, also asymptomatic at detection, showed slightly elevated citrulline levels (55 µmol/L). All these patients had normal plasma ammonia concentration at diagnosis.

### 3.2. Unscreened Cohort

The unscreened cohort included 53 patients (32 males and 21 females): 28 ASS1D (52.8%), 16 ASLD (30.2%), 7 ARG1D (13.2%), and 2 citrin deficiency (3.8%). In this group, the median age at diagnosis was 9.12 days (IQR 4 days–2.9 years), and the median follow-up time was 11.5 years (IQR 4.0–18.6 years).

### 3.3. Comparative Outcome

The mortality rate was 2.5% among patients diagnosed through newborn screening, compared with 15.1% among unscreened patients (OR 0.14; 95% CI 0.02–1.20; *p* = 0.07). A total of 15% of screened patients (3 ASLD and 3 ASS1D) showed neurological involvement, compared with 66% of those who presented with symptoms (OR 0.09; 95% CI 0.03–0.26; *p* < 0.001). The mean number of admissions due to decompensation and hyperammonemia was 0.10/year, lower than that observed in patients who presented with symptoms (0.37/year; *p* = 0.014). Liver transplantation was performed in 3 patients (7.5%) in the NBS cohort, compared with 13 patients in the unscreened cohort (24.5%) (OR 0.25; 95% CI 0.07–0.95; *p* = 0.016). In the screened cohort, two transplants were indicated because of clinical necessity (Cases 33 and 40), whereas one was undertaken as a preventive strategy (Case 4).

The two patients diagnosed with ARG1D in the screening cohort are alive and have no neurological symptoms at their current ages of 6 and 15 years, in contrast to the 7 cases diagnosed after symptom onset (*p* = 0.003). Both patients are receiving a protein-restricted diet; one of them, who was also being treated with glycerol phenylbutyrate, has recently initiated enzyme replacement therapy.

Table 2 summarizes the novel genetic variants identified in the newborn screening cohort for UCDs. The table details the corresponding nucleotide and amino acid changes and reports the classification of each variant according to ACMG criteria, as determined using two independent variant interpretation platforms. The *ASS1* variant c.365G>T (p.Gly122Val) was identified in homozygosity in Case 24, a patient who developed symptoms before receiving the newborn screening results. This patient presented with markedly elevated plasma ammonia concentration (500 µmol/L) and citrulline concentrations (1096 µmol/L) and subsequently developed cognitive impairment, suggesting that this variant is associated with a severe phenotype. In contrast, the remaining novel variants were identified in compound heterozygosity, which limits the ability to establish clear genotype–phenotype correlations.

## 4. Discussion

This retrospective, multicenter study from the Spanish Registry of UCDs provides compelling real-world evidence of the substantial clinical benefit associated with the presymptomatic diagnosis of distal UCDs through NBS. By comparing patients diagnosed through NBS with a historical cohort diagnosed after symptom onset, the study demonstrated improved survival and significantly better neurological outcomes associated with early intervention.

The most striking finding of this study is the 97.5% survival rate observed in the newborn screening cohort, in sharp contrast to the 15.1% mortality rate observed in the unscreened cohort. This result reinforces the central goal of NBS: to enable early detection and timely intervention, which may reduce the risk of an initial hyperammonemic crisis leading to death or severe irreversible neurological damage in neonatal-onset UCDs [12]. Consistent with previous reports, early identification through newborn screening and prompt initiation of therapy in ASS1D and ASLD were associated with a more favorable clinical course, including better neurocognitive outcomes and a lower incidence of severe hyperammonemic events than in symptomatically diagnosed patients [13,15].

In our cohort, screened patients showed significantly lower rates of neurological involvement, fewer hospital admissions for metabolic decompensation, and a lower need for high-risk definitive interventions such as liver transplantation. These findings support existing evidence that early detection and treatment of distal UCDs may prevent metabolic decompensation and long-term neurocognitive impairment, even in milder phenotypes [11,13].

It should be acknowledged that newborn screening cannot anticipate all early clinical presentations, particularly in severe neonatal-onset forms. In our cohort, seven patients (17.5%) had already developed symptoms by the time screening results became available. Nevertheless, most screened patients were identified presymptomatically, underscoring the overall effectiveness of NBS in this setting. Importantly, even in symptomatic cases, newborn screening results contributed to guiding the diagnostic work-up and facilitating early etiological confirmation.

The patient distribution and biochemical data suggest that the NBS protocol successfully identified a range of disease severities. The high proportion of ASS1D cases in the screened cohort (72.5%) and the finding that 62% of these patients had DBS citrulline levels below 200 µmol/L are noteworthy. This indicates that sensitive MS/MS cutoffs are essential for detecting mild or potentially late-onset cases that would otherwise be missed. The observed negative correlation between NBS citrulline levels and fibroblast ASS1 enzyme activity supports the use of citrulline in DBS as an informative biomarker of residual enzyme function, helping to distinguish severe neonatal presentations at risk of early decompensation and death from milder or moderate phenotypes, and thereby informing early management and short-term clinical decision-making, even when elevations are only moderate [11,16]. The identification of a substantial proportion of ASS1D cases with only moderately elevated DBS citrulline levels, despite interlaboratory variability in biochemical cutoffs, underscores the importance of cutoff selection for accurate and equitable case detection.

In screened cohorts, ASS1D encompasses a broad spectrum of disease severity, including mild phenotypes that may remain asymptomatic throughout life in the absence of treatment. This phenotypic heterogeneity poses important clinical challenges, particularly in determining the most appropriate individualized therapy, as both overtreatment in milder forms and undertreatment in more severe cases should be avoided. These mild phenotypes also raise important ethical and clinical questions regarding the balance between early preventive intervention and the potential risk of medicalizing individuals who may remain asymptomatic throughout life. In this context, stratification based on enzymatic activity and molecular data may help guide individualized therapeutic decision-making and optimize clinical management [5,11,16,17,18]. Furthermore, prolonged follow-up is required to better define the long-term clinical course of these milder forms. Recently, expert recommendations have been published to support clinicians in the management of patients with mild distal UCD phenotypes identified through NBS [11].

For patients with ASLD, long-term follow-up remains essential, as cognitive impairment is frequently observed despite early diagnosis and metabolic control. This suggests that factors beyond acute hyperammonemia, such as the neurotoxic effects of argininosuccinic acid accumulation and alterations in nitric oxide metabolism, may contribute to neurological outcomes [5,19]. Consequently, early identification through NBS should be complemented by sustained neurodevelopmental monitoring to better characterize and address long-term neurological sequelae in these patients.

ARG1D, which typically presents later in childhood with progressive neurological deterioration, showed a contrasting pattern in this cohort. The two patients identified through newborn screening (currently aged 6 and 15 years) have remained asymptomatic on a protein-restricted diet, with one also receiving glycerol phenylbutyrate, whereas patients in the historical unscreened cohort developed neurological manifestations. Notably, enzyme replacement therapy has recently received regulatory approval in the European Union for the treatment of ARG1D [20].

Together, these findings highlight the clinical value of presymptomatic identification and early management in disorders characterized by cumulative neurological damage over time, such as ARG1D and other distal UCDs [1,21].

With respect to the genetic data, our results delineate the range of newly identified variants detected through newborn screening for UCDs and indicate a predominance of variants of uncertain significance (VUSs). This high proportion of VUSs likely reflects both the marked genetic heterogeneity of UCDs and the limited availability of functional evidence and population-level data for rare variants identified in population-based screening programs. Discrepancies between Franklin and VarSome classifications reflect differences in evidence weighting and further highlight the challenges of variant interpretation in the newborn screening setting [22]. Together, these findings support the need for continued data sharing, longitudinal clinical follow-up, and functional studies to refine variant classification and improve the clinical utility of genomic information in newborn screening for UCDs.

As a retrospective, multicenter study, several limitations must be acknowledged. The comparison between a contemporary NBS cohort and a historical unscreened cohort introduces potential temporal and ascertainment biases. Improvements over time in neonatal intensive care, metabolic management, and the availability of therapeutic options may have contributed to differences in clinical outcomes. In particular, advances in neonatal intensive care, ammonia scavenger therapies, nutritional management, liver transplantation strategies, and specialized metabolic care networks over recent decades may have positively influenced outcomes independently of newborn screening. Therefore, the magnitude of benefit attributable exclusively to NBS should be interpreted with caution. In addition, the unscreened cohort spans a longer and earlier diagnostic period, which may partially account for the observed differences in mortality and morbidity compared with a truly contemporary control group. Furthermore, although registry-based data are invaluable for the study of rare diseases, they are inherently subject to variability in data collection practices and therapeutic protocols across participating centers. Finally, the shorter follow-up duration of the NBS cohort may underestimate late-onset complications, particularly in distal UCDs such as ARG1D and ASLD, underscoring the need for continued long-term follow-up. Because many screened patients are still children, some late-onset manifestations or subtle neurocognitive deficits may not yet be clinically evident at the time of analysis. In addition, several subgroup analyses were based on a limited number of patients. Although these findings are clinically informative and consistent with current knowledge, they should be interpreted with caution and confirmed in larger national or international cohorts with longer follow-up.

Furthermore, ongoing analyses of the Spanish UCD registry, including phenotype–genotype correlations, will be essential to personalize treatment and more accurately predict severity in patients detected by NBS.

In summary, data from the Spanish UCD Registry provide compelling real-world evidence supporting the inclusion of UCDs in national newborn screening programs. The demonstrated benefits, including a 97.5% survival rate, substantially reduced neurological involvement, and a lower need for major therapeutic interventions, indicate that early detection is associated with substantially improved clinical outcomes. These findings further underscore the need for harmonized biochemical cutoffs and coordinated long-term follow-up strategies to ensure optimal management of patients identified through newborn screening.

## 5. Conclusions

Patients with distal UCDs diagnosed through newborn screening show a significantly more favorable clinical course than those diagnosed after symptom onset, as reflected by a 97.5% survival rate, significantly lower rates of neurological involvement, and a lower frequency of hospitalizations due to metabolic decompensation and hyperammonemia. The biochemical profile of screened patients suggests that a substantial proportion have milder or attenuated disease forms, which, together with presymptomatic diagnosis and early initiation of treatment, likely contribute to the improved outcomes observed. Taken together, these findings provide real-world evidence supporting consideration of the inclusion of distal UCDs in national newborn screening programs and their incorporation into the common newborn screening portfolio of the Spanish National Health System.

## Figures and Tables

**Table 1:** Baseline clinical, biochemical and genetic characteristics of the newborn screening cohort.

Case No.	Age at Definitive Diagnosis (Days)	Symptoms at NBS Detection (Yes/No)	Plasma Ammonia at Diagnosis (µmol/L)	Citrulline-DBS (µmol/L)	Arginine-DBS (µmol/L)	Gene	Mutation 1: Nucleotide Change	Mutation 2: Nucleotide Change	Neurologic Symptoms at Follow-Up (Yes/No)
1	31	No	17	83.0	9.87	*ASS1*	c.1173C>A	c.787G>A	No
2	53	No	38	195.8	9.69	*ASS1*	c.919C>T	c.1168G>A	No
3	32	No	80	59.0	8.91	*ASS1*	c.970G>A	c.271A>G	No
4	11	No	190	563.0	5.00	*ASS1*	c.256C>T	c.256C>T	No
5	11	No	64	104.0	2.50	*ASS1*	c.919C>T	c.496-1766_597+732delinsGC	No
6	11	No	52	140.0	3.70	*ASS1*	c.919C>T	c.496-1766_597+732delinsGC	No
7	18	No	103	509.0	11.00	*ASS1*	c.970G>A	c.1168G>A	No
8	15	No	64	42.6	10.60	*ASS1*	c.19G>A	c.860G>T	No
9	18	No	59	69.0	15.00	*ASS1*	c.598-757G>A	c.598-757G>A	No
10	98	No	172	864.0	53.00	*ASS1*	c.836G>A	c.836G>A	No
11	18	No	NA	210.0	27.10	*ASS1*	c.53C>T	c.1087C>T	No
12	44	No	41	68.6	NA	*ASS1*	c.437G>A	c.919C>T	No
13	47	No	37	99.0	NA	*ASS1*	c.1168G>A	c.1003C>T	No
14	31	No	20	163.3	NA	*ASS1*	c.1003C>T	c.537G>A	No
15	24	No	35	82.3	NA	*ASS1*	c.598-757G>A	c.598-757G>A	No
16	24	No	33	65.9	NA	*ASS1*	c.598-757G>A	c.598-757G>A	No
17	49	No	124	65.4	11.67	*ASS1*	c.836G>A	To be determined	No
18	83	No	NA	134.1	14.55	*ASS1*	c.1003C>T	c.1168G>A	No
19	26	No	65	72.2	1.60	*ASS1*	c.113T>C	c.352G>A	No
20	31	No	74	65.0	1.90	*ASS1*	c.773+4A>C	c.773+4A>C	No
21	7	No	55	425.3	9.27	*ASS1*	c.1118_1123del	c.1168G>A	No
22	37	No	30	155.0	9.48	*ASS1*	c.[206T>C;808G>C]	c.805G>A	No
23	10	No	50	383.0	12.10	*ASS1*	c.1168G>A	c.535T>C	No
24	15	Yes	500	1096.0	20.40	*ASS1*	c.365G>T	c.365G>T	Yes
25	15	Yes	1500	832.8	8.17	*ASS1*	c.537G>A	c.537G>A	Yes
26 †	2	Yes	1376	903.8	38.46	*ASS1*	c.470G>A	c.470G>A	-
27	6	Yes	217	218.2	NA	*ASS1*	c.1168G>A	c.919C>T	No
28	14	No	56	128.0	NA	*ASS1*	c.1003C>T	c.1168G>A	No
29	3	Yes	NA	922.0	NA	*ASS1*	c.1168G>A	c.1168G>A	Yes
30	49	No	18	211.0	NA	*ASL*	c.446+1G>A	c.622C>T	Yes
31	14	No	60	116.0	9.00	*ASL*	c.1135C>T	c.1367G>A	No
32	16	No	50	22.0	2.99	*ASL*	c.35G>A	c.35G>A	No
33	18	Yes	316	210.0	15.00	*ASL*	c.539T>G	c.1143+1G>T	Yes
34	29	No	39	43.8	NA	*ASL*	c.1279G>A	c.617G>T	No
35	53	No	50	62.3	NA	*ASL*	c.437G>A	c.1153C>T	No
36	39	No	31	90.7	NA	*ASL*	c.532G>A	c.532G>A	No
37	11	Yes	147	271.8	19.00	*ASL*	c.209T>C	c.637C>T	Yes
38	14	No	50	17.6	196.00	*ARG1*	c.404C>T	c.181G>A	No
39	11	No	63	16.0	103.00	*ARG1*	c.913G>A	c.742G>C	No
40	57	No	40	55.0	8.50	*SLC25A13*	c.1781G>A	c.1781G>A	No

DBS: dried blood spot. NA: not available. The cut-off (upper limit) for citrulline in DBS ranges from 27.07 to 38.24 µmol/L, depending on the laboratory; for arginine in DBS, the cut-off ranges from 29.85 to 42.78 µmol/L. † Exitus at neonatal onset.

**Table 2:** Novel genetic variants identified in the newborn screening cohort for distal urea cycle disorders (UCDs) and their ACMG classification.

Case No.	Diagnosis	Presence of Symptoms at NBS Detection (Yes/No)	Mutation: Nucleotide Change	Mutation: Amino Acid Change	Franklin ACMG Classification	VarSome ACMG Classification
8	ASS1D	No	c.860G>T	p.Gly287Val	Likely Pathogenic PM2 PM1 PP3 PP2	VUS PM1 PM2
14/25	ASS1D	No/Yes	c.537G>A	p.Trp179*	Pathogenic PVS1 PM2 PP5	Pathogenic PVS1 PP5 PM2
19	ASS1D	No	c.113T>C	p.Ile38Thr	VUS PP3 PM2 PP2	VUS PM2 PP2
21	ASS1D	No	c.1118_1123del	p.Glu373_Leu374del	VUS PM2 PM4 PM1	VUS PM4 PM2
24	ASS1D	Yes	c.365G>T	p.Gly122Val	VUS PM2 PP3 PM1 PP2	VUS PM1 PP3 PM2
30	ASLD	No	c.622C>T	p.Pro208Ser	Likely Pathogenic PM2 PM5 PM1 PP3 PP2	VUS PM5 PM2 BP4
34	ASLD	No	c.1279G>A	p.Val427Met	VUS PM2 PP3 PM1 PP2	VUS PM1 PM2
38	ARG1D	No	c.181G>A	p.Asp61Asn	VUS PM2 PP2	VUS PM2 PP2
39	ARG1D	No	c.742G>C	p.Val248Leu	VUS PP3 PM2 PP2	VUS PM2 PP2 PP3

ASS1D: Argininosuccinate synthetase deficiency; ASLD: Argininosuccinate lyase deficiency; ARG1D: Arginase 1 deficiency; ACMG: American College of Medical Genetics and Genomics; VUS: variant of uncertain significance. Variant classification was performed according to ACMG guidelines at the time of publication. Criteria codes shown correspond to those applied during variant interpretation. Novel variants are defined as variants not previously reported in the literature or major public variant databases at the time of publication. Nucleotide and amino acid changes are described according to Human Genome Variation Society (HGVS) nomenclature, based on the corresponding reference transcript.

## Data Availability

The data supporting the findings of this study are not publicly available due to ethical and privacy restrictions and the need to protect participant confidentiality.

## Supplementary Materials

The following supporting information can be downloaded at: https://www.mdpi.com/article/10.3390/ijns12020044/s1, Table S1: Baseline demographic, clinical, biochemical, enzymatic and genetic characteristics of the newborn screening cohort.

## Abbreviations

The following abbreviations are used in this manuscript:

UCDs	Urea Cycle Disorders
NBS	Newborn screening
MS/MS	Tandem Mass Spectrometry
ASS1D	Argininosuccinate synthetase deficiency (also called Citrullinemia Type I)
ASLD	Argininosuccinate lyase deficiency
ARG1D	Arginase deficiency
AECOM	Spanish Association of Inborn Errors of Metabolism
IQR	Interquartile Range
DBS	Dried blood spot
ACMG	American College of Medical Genetics and Genomics
VUS	Variant of uncertain significance
