# Supplementary material for: Health Outcomes of Patients with Distal Urea Cycle Disorders Detected by Newborn Screening: Data from the Spanish National Registry
Source: Int J Neonatal Screen. 2026 Jun 18;12(2):44. doi: 10.3390/ijns12020044 (PMC13300526; doi:10.3390/ijns12020044)
Supplement: Supplementary file 1 [file IJNS-12-00044-s001.zip › IJNS-4160926-supplementary.pdf]

Supplementary Material

1. Supplementary Table S1

Table S1. Baseline demographic, clinical, biochemical, enzymatic and genetic characteristics of the newborn screening cohort.

Case Nº	Sex	Parents' country of origin	Age at definitive diagnosis (days) Symptoms at NBS detection (Yes/No)	Ammonia at diagnosis (µmol/L)	CITRULLINE-DBS (µmol/L)	ARGININE-DBS (µmol/L)	CITRULLINE-Plasma at diagnosis (µmol/L)	ARGININE-Plasma at diagnosis (µmol/L)	Enzymatic activity	Gene	Mutation 1: Nucleotide change	Mutation 2: Nucleotide change	Mutation 1: Amino acid change	Mutation 2: Amino acid change	Neurologic symptoms at follow up (Yes/No)
1	F	Venezuela/Dominican Republic	31<br>No	17	83,0	9,87	171	61	38%	*ASS1*	c.1173C>A	c.787G>A	p.Phe391Leu	p.Val263Met	No
2	F	Spain	53<br>No	38	195,8	9,69	288	99	NA	*ASS1*	c.919C>T	c.1168G>A	p.Arg307Cys	p.Gly390Arg	No
3	M	Spain	32<br>No	80	59,0	8,91	132	65	25,6%	*ASS1*	c.970G>A	c.271A>G	p.Gly324Ser	p.Thr91Ala	No
4	M	Spain	11<br>No	190	563,0	5,00	1555	9	4,8%	*ASS1*	c.256C>T	c.256C>T	p.Arg86Cys	p.Arg86Cys	No
5	F	Spain	11<br>No	64	104,0	2,50	136	35	16%	*ASS1*	c.919C>T	c.496-1766_597+732delinsGC	p.Arg307Cys	p.?	No
6	M	Spain	11<br>No	52	140,0	3,70	134	41	NA	*ASS1*	c.919C>T	c.496-1766_597+732delinsGC	p.Arg307Cys	p.?	No
7	M	Spain	18<br>No	103	509,0	11,00	3397	17	3%	*ASS1*	c.970G>A	c.1168G>A	p.Gly324Ser	p.Gly390Arg	No
8	M	Spain	15<br>No	64	42,6	10,60	122	63	NA	*ASS1*	c.19G>A	c.860G>T	p.Val7Met	p.Gly287Val	No
9	M	Morocco	18<br>No	59	69,0	15,00	112	74	30%	*ASS1*	c.598-757G>A	c.598-757G>A	p.Asn200*	p.Asn200*	No
10	M	Senegal	98<br>No	172	864,0	53,00	1102	NA	NA	*ASS1*	c.836G>A	c.836G>A	p.Arg279Gln	p.Arg279Gln	No
11	F	Spain	18<br>No	NA	210,0	27,10	275	53	NA	*ASS1*	c.53C>T	c.1087C>T	p.Ser18Leu	p.Arg363Trp	No
12	M	Colombia/Spain	44<br>No	41	68,6	NA	85	86	NA	*ASS1*	c.437G>A	c.919C>T	p.Arg146Lys	p.Arg307Cys	No
13	M	Spain	47<br>No	37	99,0	NA	74	NA	NA	*ASS1*	c.1168G>A	c.1003C>T	p.Gly390Arg	p.Arg335Cys	No

14	F	Spain	31<br>No	20	163,3	NA	118	19	NA	*ASS1*	c.1003C>T	c.537G>A	p.Arg335Cys	p.Trp179*	No
15	F	Morocco	24<br>No	35	82,3	NA	167	94	NA	*ASS1*	c.598-757G>A	c.598-757G>A	p.Asn200*	p.Asn200*	No
16	F	Morocco	24<br>No	33	65,9	NA	134	105	NA	*ASS1*	c.598-757G>A	c.598-757G>A	p.Asn200*	p.Asn200*	No
17	F	Gambia	49<br>No	124	65,4	11,67	122	62	NA	*ASS1*	c.836G>A	To be determined	p.Arg279Gln	To be determined	No
18	F	Spain	83<br>No	NA	134,1	14,55	85	59	NA	*ASS1*	c.1003C>T	c.1168G>A	p.Arg335Cys	p.Gly390Arg	No
19	M	Morocco	26<br>No	65	72,2	1,60	126	119	NA	*ASS1*	c.113T>C	c.352G>A	p.Ile38Thr	p.Ala118Thr	No
20	F	China	31<br>No	74	65,0	1,90	187	113	NA	*ASS1*	c.773+4A>C	c.773+4A>C	p.?	p.?	No
21	M	Spain	7<br>No	55	425,3	9,27	833	49	NA	*ASS1*	c.1118_1123del	c.1168G>A	p.Glu373Leu374del	p.Gly390Arg	No
22	M	Peru	37<br>No	30	155,0	9,48	142	304	NA	*ASS1*	c.[206T>C;808G>C]	c.805G>A	p.[Val69Ala;<br>Glu270Gln]	p.Val269Met	No
23	M	Spain/ Cyprus	10<br>No	50	383,0	12,10	565	17	NA	*ASS1*	c.1168G>A	c.535T>C	p.Gly390Arg	p.Trp179Arg	No
24	F	Spain	15<br>Yes	500	1096,0	20,40	2025	8	NA	*ASS1*	c.365G>T	c.365G>T	p.Gly122Val	p.Gly122Val	Yes
25	M	Morocco	15<br>Yes	1500	832,8	8,17	1645	50	NA	*ASS1*	c.537G>A	c.537G>A	p.Trp179*	p.Trp179*	Yes
26†	F	Spain	2<br>Yes	1376	903,8	38,46	1865	23	NA	*ASS1*	c.470G>A	c.470G>A	p.Arg157His	p.Arg157His	-
27	F	Spain	6<br>Yes	217	218,2	NA	190	40	NA	*ASS1*	c.1168G>A	c.919C>T	p.Gly390Arg	p.Arg307Cys	No
28	M	Spain	14<br>No	56	128,0	NA	108	60	NA	*ASS1*	c.1003C>T	c.1168G>A	1168G>A<br>p.Arg335Cys	p.Gly390Arg	No
29	M	Morocco	3<br>Yes	NA	922,0	NA	NA	NA	NA	*ASS1*	c.1168G>A	c.1168G>A	p.Gly390Arg	p.Gly390Arg	Yes
30	M	Spain	49<br>No	18	211,0	NA	138	NA	2,8%	*ASL*	c.446+1G>A	c.622C>T	p.?	p.Pro208Ser	Yes
31	F	Spain	14<br>No	60	116,0	9,00	88	NA	33%	*ASL*	c.1135C>T	c.1367G>A	p.Arg379Gly	p.Arg456Gln	No
32	M	Spain	16	50	22,0	2,99	34	23	NA	*ASL*	c.35G>A	c.35G>A	p.Arg12Gln	p.Arg12Gln	No

			No												
33	F	Spain	18<br>Yes	316	210,0	15,00	216	40	1,4 (RV 2,7-11,3 umol urea/h x gHb)	*ASL*	c.539T>G	c.1143+1G>T	p.Leu180Arg	p.?	Yes
34	F	Spain	29<br>No	39	43,8	NA	40	92	NA	*ASL*	c.1279G>A	c.617G>T	p.Val427Met	p.Gly206Val	No
35	M	Spain	53<br>No	50	62,3	NA	51	86	NA	*ASL*	c.437G>A	c.1153C>T	p.Arg146Gln	p.Arg385Cys	No
36	F	Morocco	39<br>No	31	90,7	NA	206	3	NA	*ASL*	c.532G>A	c.532G>A	p. Val178Met	p. Val178Met	No
37	F	Spain	11<br>Yes	147	271,8	19,00	938	19	NA	*ASL*	c.209T>C	c.637C>T	p.Val70Ala	p.Arg213Ter	Yes
38	M	Spain	14<br>No	50	17,6	196,00	19	196	0,43%	*ARG1*	c.404C>T	c.181G>A	p.Thr135Ile	p.Asp61Asn	No
39	F	Spain	11<br>No	63	16,0	103,00	17	149	65 (RV 3741-7805) µmol urea/h xg Hb)	*ARG1*	c.913G>A	c.742G>C	p.Gly305Arg	p.Val248Leu	No
40	M	Spain	57<br>No	40	55,0	8,50	500	372	NA	*SLC25 A13*	c.1781G>A	c.1781G>A	p.Gly594Asp	p.Gly594Asp	No

DBS: dried blood spot. NA: not available. RV: reference values. Enzymatic activity is expressed as a percentage (%) relative to the healthy control or as absolute units, depending on the method employed, reference ranges varied between laboratories. The cut-off (upper limit) for citrulline in DBS ranges from 27.07 to 38.24 µmol/L, depending on the laboratory; for arginine in DBS, the cut-off ranges from 29.85 to 42.78 µmol/L. Indicative plasma reference values for citrulline (19 ± 9 µmol/L) and arginine (61 ± 27 µmol/L) during the first month of life; reference ranges varied between laboratories. †Exitus at neonatal onset.
